# Next-Generation Sequencing Technologies and Neurogenetic Diseases

**DOI:** 10.3390/life11040361

**Published:** 2021-04-19

**Authors:** Hui Sun, Xiao-Rong Shen, Zi-Bing Fang, Zong-Zhi Jiang, Xiao-Jing Wei, Zi-Yi Wang, Xue-Fan Yu

**Affiliations:** Department of Neurology and Neuroscience Center, The First Hospital of Jilin University, Changchun 130021, China; docsunhui@163.com (H.S.); shenxr0713@163.com (X.-R.S.); bingbingaayy@163.com (Z.-B.F.); Jiangzz20@jlu.edu.cn (Z.-Z.J.); weixx16@jlu.edu.cn (X.-J.W.); wangziyi@163.com (Z.-Y.W.)

**Keywords:** next generation sequencing, neurogenetics, rare disorders, Charcot–Marie–Tooth disease, spinocerebellar ataxias, epilepsy

## Abstract

Next-generation sequencing (NGS) technology has led to great advances in understanding the causes of Mendelian and complex neurological diseases. Owing to the complexity of genetic diseases, the genetic factors contributing to many rare and common neurological diseases remain poorly understood. Selecting the correct genetic test based on cost-effectiveness, coverage area, and sequencing range can improve diagnosis, treatments, and prevention. Whole-exome sequencing and whole-genome sequencing are suitable methods for finding new mutations, and gene panels are suitable for exploring the roles of specific genes in neurogenetic diseases. Here, we provide an overview of the classifications, applications, advantages, and limitations of NGS in research on neurological diseases. We further provide examples of NGS-based explorations and insights of the genetic causes of neurogenetic diseases, including Charcot–Marie–Tooth disease, spinocerebellar ataxias, epilepsy, and multiple sclerosis. In addition, we focus on issues related to NGS-based analyses, including interpretations of variants of uncertain significance, de novo mutations, congenital genetic diseases with complex phenotypes, and single-molecule real-time approaches.

## 1. Introduction

According to the OMIM database, a wide variety of neurogenetic diseases have been discovered owing to the development of next-generation sequencing (NGS) technology. The clinical application of NGS significantly accelerated the discovery of disease-causing genes and promoted the understanding of molecular genetic mechanisms associated with hereditary diseases. Although detailed clinical phenotyping and genetic counselling are generally performed before NGS is undertaken, significant differences exist in the efficacy by which NGS can diagnose different diseases and different types of genetic variation.

The human genome consists of coding regions (exons) and non-coding regions (introns, promoters, regulatory elements, and structural elements). The sequencing of all regions is known as whole-genome sequencing (WGS). Meanwhile, whole-exome sequencing (WES) considers only coding regions (exons) for sequencing. Human exomes contain thousands of variants, including missense variants, protein-truncating variants, small indels, and large structural variants (SVs) that can span multiple genes. However, the presence of variants of unknown significance (VUS) makes it difficult for clinicians to decide whether or not to convey genetic sequencing results which could significantly increase psychological burden to patients, [1,2].

Currently, NGS technologies are limited to read lengths of approximately 150 base pairs (bp) and, thus, are incapable of identifying pathogenic expansion repeats—which can span up to thousands of bp in size [3]. Moreover, while the current gold standard for estimating repeat length is Southern blotting, it has inherent limitations including the precision of size estimation [4]. Meanwhile, third-generation sequencing has the potential to identify repeat expansion disorders—such as Friedreich ataxia, spinocerebellar ataxias (SCA), Alzheimer’ s disease, and Frontotemporal Dementia (FTD)—which are often missed by current NGS platforms.

Due to the complexity of genetic diseases, genetic testing is often employed as a last resort for patients who have visited multiple hospitals or doctors yet remain undiagnosed. Meienberg suggested that gene panels related to the patients’ phenotype could be used as an inexpensive and rapid first-tier test to address these issues. If the associated results are negative, WES or WGS can be considered as second-tier testing platforms [5]. In this review, we compare the differences between several sequencing methods and review NGS -identified genetic causes of neurogenetic diseases, including Charcot–Marie–Tooth disease (CMT), spinocerebellar ataxias (SCA), epilepsy, and multiple sclerosis (MS), introducing novel pathogenic mutations recently discovered. VUS, de novo mutations (DNMs), congenital genetic diseases with complex phenotypes, and single-molecule real-time (SMRT) sequencing are also discussed.

## 2. NGS Tools

### 2.1. Whole-Exome Sequencing

WES employs NGS platforms, such as Illumina, to sequence the protein-coding regions of the genome. Although initial sequencing and analysis of the human genome revealed that less than 2% of the genome comprises exons, approximately 85% of the DNA variations responsible for highly penetrant genetic diseases lie in this small fraction of the genome [6,7]. Currently, WES is the most commonly used mainstream sequencing method in clinical applications due to its low associated cost and turnaround time, compared with WGS.

For example, WES was applied to advance the current understanding regarding the genetic basis of amyotrophic lateral sclerosis (ALS), characterized by motor neuron degeneration. Mutations in the superoxide dismutase 1 (SOD1) gene were the first genetic mutations linked to ALS in 1993 [8]. The mutant SOD1 protein acquires a toxic function independent of its normal enzyme activity, while the expression of SOD1 mRNA in the cerebrospinal fluid serves as an indicator of disease severity in patients with ALS [6,9]. Since 2014, seven new genes associated with ALS have been identified by WES: MATR3, CHCHD10, TBK1, TUBA4A, NEK1, C21orf2, and CCNF [10]. Mutated TUBA4A interferes with the formation of microtubules in the cytoskeleton, indicating that therapeutic agents capable of enhancing the cytoskeleton may prevent, or even reverse, disease progression [11]. Thus, WES facilitated landmark changes in the management and treatment of diseases based on the identification of causal genetic factors. With further development of sequencing technology, the diagnostic rate across diverse clinical laboratories increased to 65.52% [7]. However, the overall diagnosis rate remains low, primarily due to challenges associated with the detection of pathogenic mutations, which may be classified as a VUS or appear within the noncoding region, thus escaping capture by WES. Meanwhile, mitochondrial DNA (mt-DNA) can be analysed from WES data in a holistic approach [12]. However, since the mt-DNA can vary between tissue types, likely due to differing energy demands, a negative result in one tissue type does not preclude the presence of mt-DNA variants in other tissues. Therefore, when selecting WES as a tool to identify the molecular basis of neurogenetic diseases, alternative genetic factors should not be ruled out prematurely. In fact, it is possible to obtain valuable genetic information by combining WES results with patient clinical data while employing other genetic sequencing methods, if necessary.

### 2.2. Whole-Genome Sequencing

**Figure 1:**
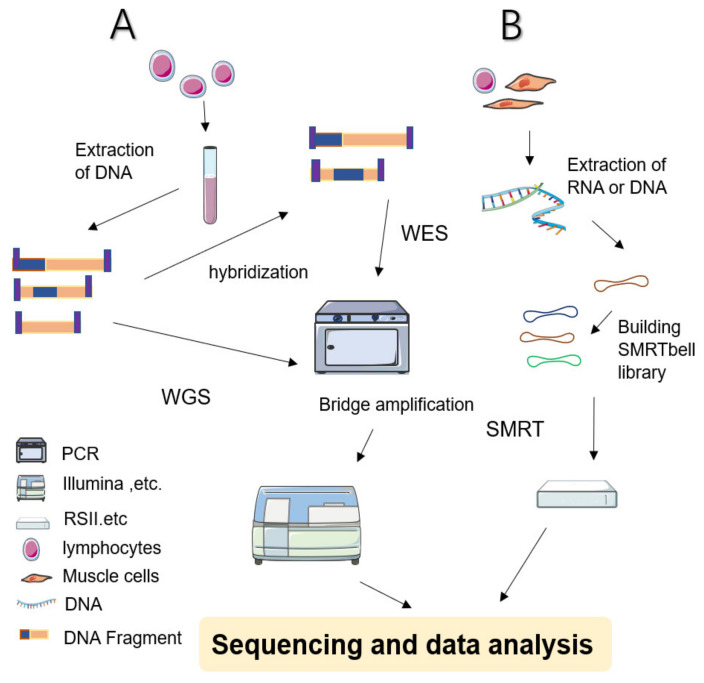
Simplified schematic diagram of NGS and SMRT. (**A**) WES: Sample preparation begins with extracted and purified DNA which is transposed into fragments. Adapters are tagged before adding motifs. Hybridization is then allowed in the flow cell. After bridge amplification, the Illumina system finally produces the first read. By contrast, WGS does not require hybridized fragments and is ready to be sequenced immediately once the library has been prepared. (**B**) SMRT: Library preparation begins with a DNA circular structure. When the polymerase encounters a strand of nucleotides containing modifications, the interpulse duration is be delayed. SMRT has two sequencing modes: circular consensus sequencing (CCS) and continuous long read (CLR) sequencing. Because CCS can scroll and copy the same segment along the circular DNA to eliminate errors, its accuracy is higher than 99%. The advantage of CLR is that it can handle longer reads.

During WGS approaches, DNA is extracted from cell sources—including peripheral blood leukocytes—and cut into several pieces before being linking with engineered DNA to be sequenced (Figure 1 shows a simplified workflow for NGS). The sequencing results are subjected to sophisticated computerized analysis and careful comparison is made with genomic reference sequences (in related databases) to obtain detailed annotation information [1,13]. WGS screens the entire genome—including coding and noncoding regions, regulatory regions, and SVs leading to copy number variations (CNVs)—facilitating the simultaneous examination of active genes and silent sequences for novel genes, variants, de novo mutations, and loci associated with specific traits [1].

An advantage of WGS is that it detects exonic single-nucleotide variants (SNVs) as well as noncoding variants, small noncoding regulatory RNAs, CNVs (including deletions, insertions, duplications, and inversions), repeat expansions, and complex chromosomal rearrangements. In mitochondrial disease, WGS has been instrumental in identifying three novel etiologic genes (COX6A1, TIMMDC1, and COQ5); the diagnosis of which relies on detection of an intronic deletion, a deep intronic variant, and a 3′-UTR duplication, respectively [14,15,16]. Moreover, within the American College of Medical Genetics and Genomics (ACMG) and RefSeq databases, WGS provides more complete coverage than WES for genes and exons [17]. In particular, WES omits approximately 0.81% of the disease-causing mutations in the Human Gene Mutation Database which are detectable by WGS. Furthermore, polymerase chain reaction (PCR)-free WGS can provide more uniform coverage of exomes compared with WGS using PCR, or WES due to the lower sensitivity of this technique to GC content, which contributes, in part, to the CNV detection capacity [5]. Additionally, de novo SNVs and CNVs, as a major cause of severe intellectual disability, are more effectively diagnosed by WGS with a rate of 42%, compared with WES at only 27% [18]. Accordingly, WGS is currently considered superior to WES in the detection of CNVs and SNVs.

Familial WGS provides information on the genetic basis of polyneuropathies with complex traits, such as Parkinson’s disease, ALS, and Alzheimer’s disease [17], with the rate of genome coverage significantly increased compared with WES. Notably, the large quantity of variants identified by WGS can restrict their accurate prioritization and the ability of WGS to provide a clear explanation or interpretation of their relative influence [19,20,21]. However, it is expected that the diagnostic rate of WGS will increase as additional noncoding variants and SVs are interpreted [20,21].

A limited number of studies have reported that the cost associated with WGS has begun to decrease, whereas the same has not been noted for WES [22]. Hence, it is predicted that WGS will become a widely accessible test for diagnostic purposes in the near future and may ultimately have greater utility than gene panels or WES in identifying pathogenic mutations.

### 2.3. Gene Panels

Gene panels initially capture a set of relevant disease-associated genes, followed by large-scale parallel sequencing [23]. Panels are particularly applicable for studying genetically heterogeneous disorders with well-defined disease-associated genes. These panels can be used to detect approximately 400 related genes for neuromuscular diseases, including congenital myasthenia, congenital myopathy, ataxias, periodic paralysis, motor neuron disorders, spastic paraplegia, Parkinson’s disease, and epilepsy, among others [17,23,24,25,26]. Owing to the low cost and high diagnostic rates, gene panel testing is a common NGS tool used in the field of neurology. For example, comprehensive analysis of the 12 currently identified genes associated with muscular dystrophy (MD) can be completed at a relatively low cost to patients [27].

Additionally, gene panels can target, and sequence, more than 80 genes associated with CMT [28]. Given the high phenotypic heterogeneity of CMT, in which distinct mutations in different genes can cause the same phenotype, panels can be restricted to specific subtypes of CMT [25,28]. Meanwhile, the diagnosis rates of certain panels, such as those for MD-related genes, are similar to those obtained with WES [29]. However, compared with WES and WGS, gene panels provide superior gene coverage, thereby reducing the likelihood of missing a mutation [30,31]. Moreover, the diagnostic rates for rare genetic CMT subtypes are increased 6- to 10-fold compared with those obtained via WES, indicating that targeted sequencing panels contribute to the identification of undiscovered pathogenic variants, thus increasing the diagnostic yield [32,33,34]. However, developing a panel relies on the establishment of known gene sequences or disease-related gene-targeting templates, making it imperative that each panel be updated in a timely manner following the publication of new associated information [35]. With further development, the commercial market can package several or even hundreds of genes to generate gene panels for diverse applications, including gene detection for precision medicine, research on hereditary diseases, early disease screening, and assessment of disease prognosis.

## 3. Application of Next-Generation Sequencing in Neurogenetic Diseases

### 3.1. Charcot–Marie–Tooth Disease

CMT encompasses Charcot–Marie–Tooth disease and the related hereditary motor neuropathy (HMN) and hereditary sensory neuropathy disorders, representing the most common group of inherited neuromuscular diseases. CMT is characterized by distal weakness, sensory loss, and a high incidence of foot deformities, including pes cavus [36]. A recent study reported that the CMT1A (PMP22), hereditary neuropathy with liability to pressure palsy/PMP22 deletion (HNPP), CMTX1 (GJB1), CMT1B (MPZ), and CMT2A (MFN2) subtypes account for 89.2% of all genetically diagnosed CMT cases [37]. Founder effect refers to the separation of small groups from a larger original population, which can lead to a random selection of certain alleles and ultimately alters the allelic frequency of the population—a process referred to as genetic drift [38]. To date, more than 100 genes with pathogenic mutations have been described in relation to CMT. The most common subtype is CMT1A, which accounts for more than 60% of diagnosed cases [39]. Genetic drift contributes to the irregular distribution of different CMT subtypes in each geographical and ethnic population worldwide. In fact, in certain instances, a founder mutation has been shown to be more prevalent. For example, in Slovakia, NDRG1 and HK1 genes, rather than CMT1A, are responsible for the majority of Roma cases in some areas [40]. Hence, the approach taken for diagnosing CMT may differ based on the specific ethnic background of the patient [28].

According to the phenotype–genotype correlation classification, Pipis et al. [28] classified CMT-associated genes based on different parts of the body affected. Clinical phenotyping is particularly important in the genomic era as identifying subtle or unexpected distinguishing features of specific genes might help elucidate the underlying genetic mechanisms. For instance, NEFL (CMT2E) mutations are reportedly associated with cerebellar syndrome; however, a novel variant (c.269A>G) was identified in a French woman who exhibited common symptoms of moderate sensorineural deafness, excluding lower limb involvement [41]. Additionally, sensorineural hearing loss is reported to be associated with GJB1. In a Chinese family, the proband with a mutation identified in the nerve-specific promoter P2 region of GJB1 (c.-170T>G) showed reversible white matter lesions in the brain, which was suggested to be associated with disruption in gap junction communication between oligodendrocytes and astrocytes, leading to the inability of these cells to regulate fluid exchange and ultimately resulting in cell oedema [42].

Symptoms of CMT with autosomal dominant (AD) inheritance are generally milder and differ from autosomal recessive (AR) types. Kim et al. [43] found that patients with AD and AR forms of CMT harbouring GDAP1 mutations showed significant differences in lower limb magnetic resonance imaging (MRI). That is, the posterior-compartment muscles were affected in AD-CMT patients, whereas AR-CMT patients primarily exhibited fatty infiltration in the anterolateral-compartment muscles. Meanwhile, combinations of multiple variants are rarely described. Nevertheless, in a family with CMT, the conditions of the mother and daughter were characterized by axonal damage, and were associated with MORC21, MFN2, and AARS1 variants [44]. MFN2 is a member of the mitochondrial transmembrane protein family, which is widely expressed by eukaryotic cells and plays an important role in mitochondrial fusion and division-controlled mitochondrial dynamic remodelling [45]. MORC2, in combination with the MFN2 variant, result in more severe phenotypes and complex clinical symptoms. The AARS1 variant was also found in healthy members of the family, suggesting it was not an independent risk factor [44]. However, the authors were unable to conclusively determine whether the AARS1 variant worsens CMT based on the presence of other causal mutations.

BAG3 mutations have been shown to be primarily associated with myofibrillar myopathy 6, CMT, and dilated cardiomyopathy 1HH [46,47]. The point mutation of c.625C>T was reported as a hotspot causing neuromuscular phenotypes focused on the Pro209 residue, whereas mutations causing dilated cardiomyopathy 1HH were distributed throughout the gene [46,48]. WGS is more valuable for patients with CMT who have genetic variants outside of exon regions. For instance, an 11-year-old girl was found to carry a maternally inherited rare variant (RV) in IGHMBP2 (c.1730T>C) that was predicted to be pathogenic; however, no variant was identified on the other allele. WGS confirmed the previously identified IGHMBP2 RV and identified a paternally inherited noncoding IGHMBP2 RV [49].

Recently, mutations in mitochondria-related genes have also been linked to CMT. A recent study showed that, in addition to the known mitochondrial RNA (ATP6, encoding a complex V subunit), CMT-related mitochondrial tRNA mutations led to a selective decrease in tRNA levels. This change affects the addition of valine to the growing peptide chain and the function of mitotic ribosomes during translation [50]. Therefore, strategies for increasing mitochondrial tRNA production may alleviate the effects of this type of variation.

### 3.2. Spinocerebellar Ataxias

SCAs generally refer to a group of ataxias with AD inheritance. Additionally, a portion of AR ataxias is designated as SCAR [51]. There are 48 subtypes of SCAs with 36 pathogenetic genes identified to date. More than 100 SCAR genes have also been revealed by NGS [52]. Genetically, SCAs are categorized as either repeat expansion or nonrepeat mutations [51]. Although there is currently no consensus on the optimum order of genetic tests for SCAs, it is recommended to first test for CAG repeat expansions due to their high prevalence. If the result is negative, gene panels and WES can then be considered according to the specific situation [51,52]. A retrospective study published in 2021 estimated the diagnostic yield in 124 SCA patients from 102 families in Italy, with a reported total diagnosis rate of 52%. AD-SCA patients had the highest diagnostic yield (64.5%) among the SCA cases, of which the most frequent subtype was SCA2, followed by SCA1, SCA3, SCA6, and SCA7 [53]. Furthermore, the genetic epidemiology of SCA, like that of any rare disease, is affected by founder effect. For instance, SCA3 is the most common subtype globally, as well as in Western Europe, but has not been identified in Poland. Interestingly, SCA1, caused by ATXN1 gene mutations, is the most prevalent subtype in Poland compared with any other country and may, thus, represent a potential founder effect [54].

Gene panels and WES have served as helpful tools for the diagnosis of AD ataxias (Table 1). SCA6 is caused by CAG-repeat expansion in exon 47 of the CACNA1A gene [55]. Specifically, Saathoff et al. [56] revealed a new pathogenic nonsense variant in CACNA1A (c.2983G>T), which exhibited segregation with the disease for other family members with cerebellar syndrome. The case indicated that this nonsense variant might cause the disease by producing a truncated protein or via nonsense-mediated mRNA decay. Further, Deng et al. [57] sequenced a three-generation Chinese family with SCA11 and identified a novel point mutation (c.3290T>C) in TTBK2. The mutation segregated with the phenotype and was predicted to cause protein damage and functional impact. Additionally, Shirafuji et al. [58] reported the first nonsense mutation in PRKCG (c.226C>T) that was suggested to cause SCA14. PRKCG encodes protein kinase C gamma (PKCγ), which was suggested to cause cell death by suppressing PKC kinase. Satoh et al. [59] investigated two Japanese families with SCA23 and identified pathogenic variants in the prodynorphin gene (PDYN). The patient with a homozygous mutation had a younger age of onset and more serious ataxic symptoms than those of the heterozygous patients in the first family. Hence, the existing hypotheses of toxic gain or loss of function may not fully explain the mechanism of SCA23. Furthermore, the authors suggested that the newly discovered PDYN variant (c.644G>A) contributes to symptom severity via a dosage effect.

SCA48 is a subtype of SCA characterized by cerebellar cognitive affective syndrome (CCAS) associated with a pathogenic variant in STUB1, which is considered to function in cognitive and emotion-related areas of the cerebellum. Genis et al. [60] hypothesized that a mutation in STUB1 causes cerebellar degeneration via loss-of-function or toxic dominant gain-of-function mechanisms. Additionally, De Michele et al. [61] studied eight patients from two Italian families and found two novel mutations in STUB1. However, in contrast to the findings of Genis et al. [60], the clinical features of these patients more closely resembled those of SCAR16 and SCA17. Although the authors speculated that the mechanism might be related to dysfunction of the autophagy pathway, they highlighted that the relationship between mutations in STUB1 and the complex phenotypes observed in SCAR16 and SCA48 remains unclear.

Compared with SCA, SCAR is rarer and more sporadic [62,63]. A recent Japanese study identified two novel compound heterozygous variants (c.667C>T and c.853del) in PMPCA. The patient presented with infancy onset and had a more severe and progressive form compared with other reported cases associated with PMPCA variants. The authors speculated that variants causing functional impairment of PMPCA and extra cross-linking in the cells may account for the severe phenotype of the proband [64]. In addition, Zagnoli-Vieira et al. [65] described an American boy with SCA23 who harboured the same TDP2 variant previously reported in three Irish siblings. Interestingly, they demonstrated the impact of the TDP2 mutation on nuclear DNA double-strand breaks using patient fibroblasts. Subsequently, Ciaccio et al. [66] reported a novel homozygous TDP2 nonsense variant (c.400C>T) in a 17-year-old girl. The cerebellar atrophy and drug resistance to antiepileptics in this patient further supported the notion that SCAR23 is a degenerative disorder, thus, amending the view purported by Zagnoli-Vieira et al. [65]. GRM1 encodes the metabotropic glutamate receptor type 1 (mGluR1), which is a transmembrane protein that is highly expressed in cerebellar Purkinje cells. A novel homozygous truncating variant in GRM1 (c.889C>T) was recently identified in a Tunisian boy. The nonsense variant was considered pathogenic as it was located in the ligand-binding domain and led to loss of mGluR1 function [67].

### 3.3. Epilepsy

More than 65 million people worldwide suffer from epilepsy with 70–80% of these cases caused by genetic factors [68]. Targeted panels or WES can provide a genetic diagnosis for up to 30% of patients with early-onset epilepsy and for approximately 25% with de novo mutations [69]. However, complex genotype–phenotype correlations make the aetiology and therapy for epilepsy difficult [70,71,72]. Demographically, a significant discovery includes positive diagnostic findings closely related to age. Childhood epilepsy, or early-onset epilepsy, typically has a higher diagnostic yield compared with adult-onset epilepsy [73,74,75], suggesting that the molecular mechanisms may differ.

Using NGS platforms, several genes encoding voltage-gated ion channels were defined as being associated with epileptic encephalopathies (EE) and developmental and epileptic encephalopathies (DEE) [76,77]. Inuzuka et al. [78] described a patient with an uncommon form of hyperkinetic focal motor seizure in EE carrying a newly discovered variant of KCNT2 which affected the putative pore-forming domain of the protein. A group of diseases characterized by monogenic inheritance and developmental disorders, designated (DEE), is a main beneficiary of NGS [79]. KCNA2 is a DEE-associated gene that encodes the voltage-gated K+ channel KV1.2 [76]. For instance, Gong et al. [80] reported the first known patient with mosaicism in KCNA2, who had two different mosaic mutation alleles at the same nucleotide in KCNA2: c.1225A>T and c.1225A>C. However, it remains unclear whether complex mosaicism is a contributor to the clinical underdiagnosis of KCNA2-related encephalopathy.

Given that the aetiology of most EE is ambiguous, researchers have suggested that genetic mutations may be responsible for some of these cases. Several EE-associated genes have also been identified. An epilepsy panel revealed a new de novo pathogenic heterozygous mutation of the γ-aminobutyric acid type A (GABAA) receptor γ2 subunit gene (GABRG2; c.917C>T) in an 11-month-old boy [81]. This newly discovered variant is situated near p.P302L, another de novo variant in GABRG2 that was identified in a patient with Dravet syndrome—a type of early-onset EE [82]. Both variants affect the transmembrane segment M2. Additionally, p.P302L was confirmed to result in GABAA gating impairment, hyperexcitability, GABAA receptor desensitization, and ultimately formation of the epilepsy phenotype [81,82]. Clinically, Sun et al. [83] investigated 205 aetiologically undetermined cases of DEE and identified four novel mutations in SZT2 in three patients, all of whom suffered from refractory epilepsy and had special MRI results. Japanese researchers also identified a de novo variation in NUS1 (c.691+1C>A) in two unrelated individuals. In contrast with other reported mutations in NUS1 associated with developmental delays, ataxia, intellectual disability, and DEE, both individuals had scoliosis. Hence, this study strongly suggests that loss-of-function variants in NUS1 that result in loss of the cis-PTase domain in the C-terminus of NgBR may be related to scoliosis and represent a new phenotype [84]. These findings expand the phenotypic spectrum of the NUS1 gene.

In genetic epilepsy, seizures are a primary symptom. Various genetic aetiologies of genetic epilepsy have been postulated, however, the pathogenic genes in most cases remain unknown [79]. SCN9A is a Nav1.7 sodium channel protein-encoding gene that is associated with genetic epilepsy and febrile seizures. Banfi et al. [85] performed a gene panel assay in a family including a male proband with genetic epilepsy. After a 2-year treatment with lamotrigine, a Brugada pattern on electrocardiogram (ECG) was observed, which can be hypothetically regarded as a drug-induced cardiac conduction abnormality. When pathogenic variants affect sodium channel expression, corresponding auxiliary investigations, including ECG, should be considered [85]. WES in a Malaysian-Chinese family with different epilepsy phenotypes identified a novel nonsynonymous substitution (c.5753C>T, p.S1918F) in SCN1A that was present in all family members with genetic generalized epilepsy (GGE). The novel mutation was presumably pathogenic, impairing the function of sodium channels by disturbing the calmodulin-associated pathway [86]. Bonzanni et al. [87] identified another novel de novo mutation in HCN1 in a patient affected by GGE, which altered neuronal discharge activity.

Approximately half of patients with intractable epilepsy accompanied by intellectual disability remain undiagnosed. In this context, NGS may provide the means for identification of etiological factors. Using NGS, Ittiwut et al. [88] identified a de novo variant (c.467A>T) in ATP6V0C in a patient with intractable epilepsy and intellectual disability, which was a conserved termination codon mutation. Meanwhile, Wu et al. suggested that NEXMIF with X-inactivation patterns might have contributed to the mild intellectual disability [89].

NGS has also played an important role in identifying the rare aetiology of some neurological diseases accompanied by epileptic seizures. For instance, cerebral folate deficiency (CFD) is a neuropsychiatric disorder with characteristic low cerebral spinal fluid and 5-methyltetrahydrofolate (MTHF) levels. FOLR1 mutations are a rare cause of CFD, and most patients with these mutations share similar clinical phenotypes as those with other common causes, such as frequent epileptic seizures [90,91,92]. Mafi et al. [93] attributed the observed myoclonic seizures to the novel variant (c.197 G>A) in FOLR1 identified by WES (Table 1).

**Table 1:** Variants associated with neurogenetic diseases.

Disease	Ref (Year)	Country	Gene	Variant	NGS	Inheritance
CMT	Lerat et al. [41]	France	NEFL	c.269A>G	Panel	AD
	Luo et al. [42]	China	GJB1	c.-170T>G	unclear	XD
	Miressi et al. [44]	France	MORC21 Mfn2	c.568C>T c.1403G>A	Panel WES	-
	Fu et al. [46]	China	BAG3	c.625C>T	WES	AD
	Cassini et al. [49]	USA	IGHMBP2	c.1235+894C>A	WGS	AR
	Fay et al. [50]	Venezuelan	mt-tRNA	m.1661A>G	WES	MI
SCA	Saathoff et al. [56]	Germany	CACNA1A	c.2983G>T	Panel	AD
	Deng et al. [57]	China	TTBK2	c.3290T>C	WES	AD
	Shirafuji et al. [58]	Japan	PRKCG	c.226C>T	WES	AD
	Satoh et al. [59]	Japan	PDYN	c.644G>A	Panel	AD
	Genis et al. [60]	Spain	STUB1	c.823_824delCT	WES	AD
	De Michele et al. [61]	Italy	STUB2	c.97G>A c.682C>T	WES	AD
	Takahashi et al. [64]	Japan	PMPCA	c.667C>T c.853del	WES	AR
	Ciaccio et al. [66]	Italy	TDP2	c.400C>T	WES	AR
	Cabet et al. [67]	Tunisian	GRM1	c.889C>T	WES	AR
Epilepsy	Inuzuka et al. [78]	Brazil	KCNT2	c.725C>A	WES	
	Gong et al. [80]	China	KCNA2	c.1225A>T, c.1225A>C	WES	
	Komulainen et al. [81]	Finland	GABRG2	c.917C >T	Panel	
	Sun et al. [83]	China	SZT2	c.1626+1G>A, c.5772dupA, c.4209C>A, c.7307_7308insG	Panel	
	Den et al. [84]	Japan	NUS1	c.691+1C>A	WES	
	Banfi et al. [85]	Italy	SCN9A	c.319 T>C	Panel	
	Chan et al. [86]	Malaysia	SCN1A	c.5753C>T	WES	
	Bonzanni et al. [87]	Italy	HCN1	c.469C>G	Panel	
	Ittiwut et al. [88]	Thailand	ATP6V0C	c.467A>T	WES	
	Wu et al. [89]	China	NEXMIF	c.1063delC	WES	
	Mafi et al. [93]	France	FOLR1	c.197G>A	NGS	

This table shows a selection of studies published within 3 years that use next generation sequencing (NGS) as designs to identify rare variants. Ref, reference; CMT, Charcot–Marie–Tooth disease; SCA, spinocerebellar ataxias; AD, autosomal dominant; AR, autosomal recessive; XD, X-linked dominant inheritance; MI, mitochondrial inheritance.

### 3.4. Multiple Sclerosis

The application of NGS in MS currently focuses on identifying microRNAs (miRNAs), which participate in the pathogenesis of MS through various biological processes influencing immune cells in innate and adaptive immunity [94]. Researchers found that four circulating miRNA exosome sequences were differentially expressed in relapsing-remitting multiple sclerosis (RRMS) patients compared with healthy controls. These results indicated that miRNAs are expected to become a biomarker for predicting and distinguishing MS relapse [95]. Moreover, secondary progressive multiple sclerosis (SPMS) causes modest immune activation compared with RRMS. In fact, an NGS study revealed that miRNA expression declined in the CD4^+^ T cells of SPMS patients [96]. A study in 2019 confirmed this conclusion in experimental autoimmune encephalomyelitis—an animal model of MS—demonstrating that suppressed miRNAs and long noncoding RNAs (lncRNA) were consistent with alleviated symptoms following cannabidiol treatment. Additionally, class I/II human leukocyte antigen (HLA) genes contribute to an individual’s susceptibility for developing MS. A recent study analysed variants of 16 HLA genes and identified alleles associated with MS risk [97]. However, similar to other studies, an exome-sequence analysis of four multi-incident MS families did not identify individual disease-causing gene variants [98,99,100,101]. These results highlight the complex genetic aetiology of MS, and the possibility of a multigenic origin. Hence, current clinical studies have been deficient, and additional research with larger patient populations is required.

## 4. De Novo Mutations

DNMs are germline mutations that are present in most cells of an individual but are absent in the parents. DNMs are more deleterious than inherited variations because they have avoided stringent evolutionary selection [102]. DNMs can be divided into three categories: (1) those which occur during spermatogenesis or oogenesis; (2) postzygotic mutations; (3) those which occur after the separation of embryonic germline and somatic tissues. Furthermore, DNMs contain various specific mutation types, including single-nucleotide substitutions, insertions, deletions, and copy-number variants [103]. Genetic counselling involves the comprehensive assessment of a patient’s genome as the recurrence risk differs between postzygotic mutations and mutations in germ cells [104]. Additionally, confirmed somatic mosaicism in a parent carries an increased risk of recurrence after the birth of an affected child. Moreover, if the germline mosaicism of parents cannot be excluded, the risk of a second child being affected can be as high as 8.6% [105].

In adult-onset neurodegenerative disorders, DNMs can explain sporadic cases of Alzheimer’s disease, Parkinson’s disease, frontotemporal lobar degeneration spectrum disorders, ALS, and prion disorders. Trio-based whole-exome sequencing (Trio-WES) and WGS serve to improve the discovery of novel disease-causing genes [102,106,107,108,109,110]. Meanwhile, WGS/WES analysis of the affected proband together with normal parents (trio) is a common method for identifying de novo DNMs [111]. However, any detected variation should be verified by biological software or animal experiments [109]. Further, considering that candidate mutations are not necessarily known genes for diseases affecting brain development and brain function, it remains challenging to establish pathogenicity and specific disease mechanisms without additional research of each mutation individually [107,108].

## 5. Variants of Uncertain Significance

The interpretation of VUS is challenging as the pathogenicity of variants is not previously reported and is, thus, considered to be of uncertain significance. There are multiple potential reasons for the occurrence of VUS: (1) due to the wide application of gene detection technology, increasing numbers of patients undergo multigene detection resulting in significant differences between individuals; (2) a new guideline for interpreting variations requires more stringent classification of these variations to ensure the accuracy of results, which is partially responsible for the current classification of most variations as VUS. Accordingly, ACMG convened a working group in 2013—including ACMG, AMP, and a representative of the American Society of Pathologists—to review and revise the criteria and guidelines for the interpretation of sequence variation. Presently, novel sequencing (e.g., long-read sequencing) and informatics can be used to detect variations that may be difficult to detect with standard methods. Long-read sequencing techniques, such as PacBio [112] and Oxford Nanopore [113], coupled with chain-reading platform 10× genomics [114], can improve the resolution of repeat regions, large indels, and structural variations [114,115].

## 6. Phenotypic Heterogeneity: Suggesting the Road to Aetiology Exploration

In a rare and extreme condition, patients exhibit phenotypes of two congenital diseases. Thus, when confronting diseases that are difficult to diagnose, it is suggested that monism should be used to explain the etiological factors. Congenital myasthenia syndrome (CMS), comprising a group of monogenetic disorders that affect neuromuscular junction, offers a sound explanation for this condition. Among the 32 known genes in CMS, the phenotypes associated with DOK7, MUSK, DPAGT1, CHRNE, and GMPPB can coincide with muscular diseases, such as MD, limb–girdle muscular dystrophy (LMD), and myopathy [116]. Some patients with GMPPB or CHRNE mutations present with MD-like symptoms [117]. The myopathy-like clinical and pathological manifestations of CHRNE, which are involved in slow-channel congenital myasthenic syndrome, are primarily caused by calcium overload due to the delayed closure of slow ion channels [118]. In contrast to the pathway associated with CHRNE, defects in protein glycosylation caused by GMPPB lead to AChR subunits incorrectly settled, and expressed, on the surface of cells [119]. Approximately 40 genes are associated with MD and are primarily involved in extracellular matrix and basement membrane proteins [120]. GMPPB is also involved in N-glycation and O-mannose glycation pathways [121]. In the case of GMPPB, pathological changes of muscular and neuromuscular junctions can be present simultaneously, with the clinical manifestations of LMD and CMS overlapping, or concealing, each other. Therefore, in the complex background of neurogenetic diseases, the pathological mechanisms of different diseases may intersect. Hence, NGS is extremely important for diseases with more than one congenital disease phenotype, with the genes screened by WGS potentially providing insights into new mechanisms. A similar example is that of GARS, which causes distal upper limb dyspraxia and was not only found in CMT, but also in autism spectrum disorder, mitochondrial disease, and motoneuron disease [122,123,124].

## 7. Single-Molecule Real-Time Sequencing

Sanger sequencing is the most effective means to verify the accuracy of sequencing. Compared with the high cost, low throughput, and difficulty in obtaining data from Sanger sequencing, NGS provides a low-cost and high-throughput platform. NGS takes the edge synthesis and sequencing of Illumina as the mainstream, which increases the error rate of sequencing in PCR amplification. Furthermore, system bias is inevitable and the read length is short. Recently, third-generation sequencing (TGS) has emerged as an improved supplement to overcome the shortcomings of NGS. Specifically, long-read technologies have the potential to identify the ambiguous genome regions that NGS cannot explain [125]. The major representatives of TGS include single-molecule real-time (SMRT) sequencing from Pacific Biosciences (PacBio) and nanopore sequencing from Oxford Nanopore Technologies.

Owing to the characteristic long reads, TGS facilitates the exploration of molecular mechanisms associated with diseases that are caused by expansion of noncoding repeats. For example, using both SMRT and nanopore sequencing, Japanese researchers found that expansion of noncoding TTTCA repeats in an intron of SAMD12 contribute to the onset of benign adult familial myoclonic epilepsy (BAFME), an AD disorder [126]. However, nanopore sequencing may cause error reads as researchers have reported substantial variation in the repeat lengths [126]. Similarly, another study in 2019 used SMRT to demonstrate that the BAFME4 subtype is caused by insertion of the intronic TTTCA repeats in YEATS23 [127]. Thus, TGS can be used to explore the pathogenesis of neurogenetics that NGS is incapable of elucidating.

Although SMRT has been widely used in sequencing repeat expansions, its limitations cannot be ignored. In a study aimed at patients with SCA10, SMRT preferentially sequenced small repeat-sized alleles while failing to generate circular consensus sequences of expanded SCA10 repeats containing large (ATCCC) repeat interruptions. Researchers held that this was due to high GC content [128]. Hence, the constitution of repeats may influence the utility of SMRT.

In conclusion, SMRT is a vigorous technology that can detect, and characterize, the expansions of noncoding regions ignored by NGS. To date, this platform has been employed in studies focused on genetic epilepsy, neurodegenerative diseases, and Parkinson’s disease [126,127,129,130]. Further research is warranted to explore the complex molecular mechanisms underlying neurogenetic diseases, particularly single-gene diseases. With the continuous improvement of technical and biological information tools, long-read sequencing is likely to become a routine feature of the rare disease genomics tool kit.

## 8. Concluding Remarks and Future Perspectives

NGS is an integral component for delivering precise therapy options to patients. Indeed, NGS may one day replace some of the current methods used for disease diagnosis. An example of this was demonstrated by a population-based study which reported that the highest epilepsy diagnostic yield was obtained via MRI (65%), and, although WES/WGS was performed in only 26/116 cases (22%), the associated diagnostic yield reached 58% [74]. Additionally, the diagnosis of certain conditions associated with neuromyopathy, such as MD, and CMS can avoid the need for muscle biopsy by instead performing genetic testing [131]. Moreover, NGS may become a tool for early intervention or trial treatment, thereby greatly reversing disease trajectory [132,133]. As hereditary diseases account for a significant proportion of morbidity and mortality in infants, rapid whole-genome sequencing can be employed as a primary test for critically ill newborns, thus accelerating the delivery of effective treatments and reducing medical costs [133]. Meanwhile, NGS is gradually realizing accurate symptomatic treatment—perfect examples of which are advances in treatment for Walker–Warburg syndrome and late-onset Pompe disease [134]. This allows doctors to provide accurate advice on treatment options, long-term outcomes, and rehabilitation needs [135]. Selecting the correct genetic test based on cost-effectiveness, coverage area, and optimal test time ensures that clinicians can accurately assess the risk to other family members or future generations and provide genetic guidance.

## Data Availability

All data resides within the author’s premises and will be made available upon reasonable request.
